# Supplementary material for: Evolutionary shaping of human brain dynamics
Source: eLife. 2022 Oct 26;11:e80627. doi: 10.7554/eLife.80627 (PMC9662828; doi:10.7554/eLife.80627)
Supplement: Supplementary file 1. [file elife-80627-supp1.docx]

**Supplementary File 1. Table listing the names of 57 cortical regions in each hemisphere.**

| bankssts_1 | middle temporal_1 | rostral middle frontal_1 |
| --- | --- | --- |
| caudal anterior cingulate_1 | middle temporal_2 | rostral middle frontal_2 |
| caudal middle frontal_1 | parahippocampal_1 | rostral middle frontal_3 |
| cuneus_1 | paracentral_1 | superior frontal_1 |
| entorhinal_1 | parsopercularis_1 | superior frontal_2 |
| fusiform_1 | parsorbitalis_1 | superior frontal_3 |
| fusiform_2 | parstriangularis_1 | superior frontal_4 |
| inferior parietal_1 | pericalcarine_1 | superior parietal_1 |
| inferior parietal_2 | postcentral_1 | superior parietal_2 |
| inferior temporal_1 | postcentral_2 | superior parietal_3 |
| inferior temporal_2 | postcentral_3 | superior temporal_1 |
| isthmus cingulate_1 | posterior cingulate_1 | superior temporal_2 |
| lateral occipital_1 | precentral_1 | supramarginal_1 |
| lateral occipital_2 | precentral_2 | supramarginal_2 |
| lateral orbitofrontal_1 | precentral_3 | frontal pole_1 |
| lateral orbitofrontal_2 | precentral_4 | temporal pole_1 |
| lingual_1 | precuneus_1 | transverse temporal_1 |
| lingual_2 | precuneus_2 | insula_1 |
| medial orbitofrontal_1 | rostral anterior cingulate_1 | insula_2 |
